# Intelligent Beam Optimization for Light-Sheet Fluorescence Microscopy through Deep Learning

**DOI:** 10.34133/icomputing.0095

**Published:** 2024-07-04

**Authors:** Chen Li, Mani Ratnam Rai, Yuheng Cai, H. Troy Ghashghaei, Alon Greenbaum

**Affiliations:** 1 Joint Department of Biomedical Engineering, North Carolina State University and University of North Carolina at Chapel Hill, Raleigh, NC 27695, USA.; 2 Comparative Medicine Institute, North Carolina State University, Raleigh, NC 27695, USA.; 3 Department of Molecular Biomedical Sciences, North Carolina State University, Raleigh, NC 27695, USA.; 4 Bioinformatics Research Center, North Carolina State University, Raleigh, NC 27695, USA.

## Abstract

Light-sheet fluorescence microscopy (LSFM) provides the benefit of optical sectioning coupled with rapid acquisition times, enabling high-resolution 3-dimensional imaging of large tissue-cleared samples. Inherent to LSFM, the quality of the imaging heavily relies on the characteristics of the illumination beam, which only illuminates a thin section of the sample. Therefore, substantial efforts are dedicated to identifying slender, nondiffracting beam profiles that yield uniform and high-contrast images. An ongoing debate concerns the identification of optimal illumination beams for different samples: Gaussian, Bessel, Airy patterns, and/or others. However, comparisons among different beam profiles are challenging as their optimization objectives are often different. Given that our large imaging datasets (approximately 0.5 TB of images per sample) are already analyzed using deep learning models, we envisioned a different approach to the problem by designing an illumination beam tailored to boost the performance of the deep learning model. We hypothesized that integrating the physical LSFM illumination model (after passing it through a variable phase mask) into the training of a cell detection network would achieve this goal. Here, we report that joint optimization continuously updates the phase mask and results in improved image quality for better cell detection. The efficacy of our method is demonstrated through both simulations and experiments that reveal substantial enhancements in imaging quality compared to the traditional Gaussian light sheet. We discuss how designing microscopy systems through a computational approach provides novel insights for advancing optical design that relies on deep learning models for the analysis of imaging datasets.

## Introduction

Current advancements in imaging can be attributed to 2 parallel research efforts: first, the improvement of optical system design and components to achieve superior properties, such as imaging speed and depth, signal-to-noise ratio, and more [1–7]; second, the utilization of advanced postprocessing algorithms to enhance image quality and potentially streamline the data extraction phase [8–14]. The latter approach has gained popularity with the development of deep learning techniques since it avoids alterations to existing optical setups and investments in hardware, and has the ability to faithfully mimic human performance in data analysis [15–19]. There is an ongoing effort to merge these 2 research streams and jointly optimize the imaging system and the deep learning network [20–26]. This approach, which we refer to as “deep design” (DD), involves incorporating the physical model into the mathematical framework of deep learning. One of the first applications of DD in microscopy was showcased in 3-dimensional (3D) localization microscopy for densely labeled live cells [26]. This work aimed to achieve snapshot depth reconstruction under low light conditions by optimizing 2 physical phase masks in the split detection path of a standard microscope. DD was utilized to jointly optimize the physical phase masks and the accompanying neural network, which was used to detect the 3D emitters, also referred to as the image processing network. This DD approach led to a 2-fold increase in the detection rate and enhanced precision compared to state-of-the-art single-channel designs. Moreover, this work yielded superior results when compared to phase masks that were optimized using conventional optimization methods. Consequently, the DD approach, which jointly optimizes the optical components and the image processing, appears to be promising.

**Fig. 1.**
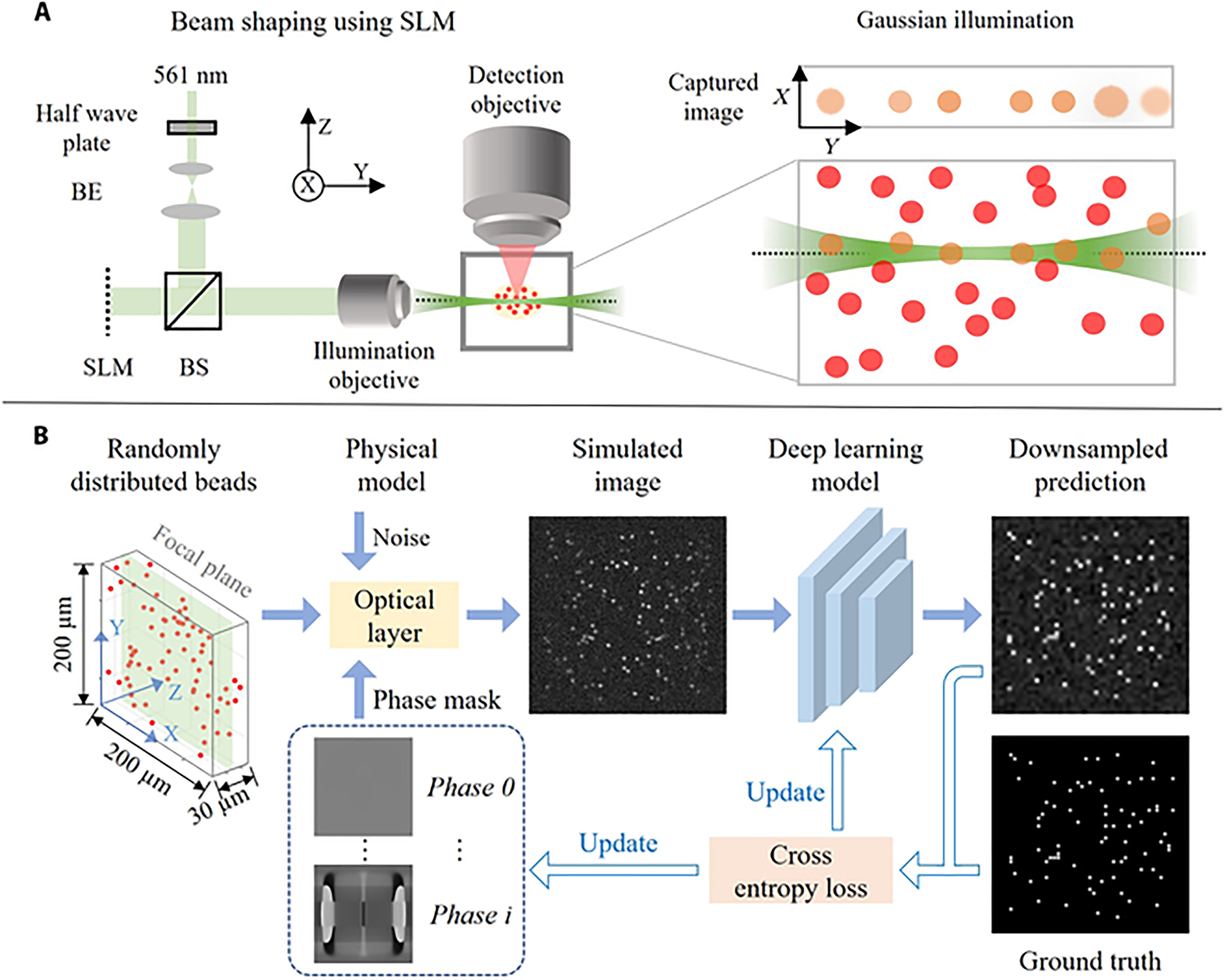
Deep design approach to improve optical sectioning and image quality in LSFM. (A) Simplified optical setup with spatial light modulator (SLM) for controlling the illumination beam. The close-up highlights a challenge encountered when employing a Gaussian beam in light-sheet fluorescence microscopy: diffraction causes the illumination beam to widen at the edges. Consequently, out-of-focus beads are unintentionally illuminated, introducing noise into the image. BS, beam splitter; BE, beam expander. (B) Joint optimization scheme. The locations of randomly distributed beads are fed into a physical optical layer to generate simulated images. The prediction network outputs a 2D downsized image to predict the positions of beads in the focal plane. The deep learning network and input phase mask are simultaneously updated based on the loss function.

Here, we will demonstrate the potential of DD in the context of light-sheet fluorescence microscopy (LSFM). LSFM has become an indispensable imaging tool in the life science community given its high acquisition speed and optical sectioning capabilities [27–32]. In LSFM, the illumination path is separated from and orthogonal to the detection path. A thin plane of light is used to illuminate a tissue in which excited fluorophores emit light that is collected by a wide-field detection system, which is oriented perpendicularly to the light-sheet illumination axis [33,34]. LSFM image quality depends on various factors. Lateral resolution is influenced by fluorescence wavelength and the numerical aperture (NA) of the detection objective, while axial resolution and signal-to-noise ratio are broadly influenced by the shape of the illumination pattern and the NA of the detection objective [32,35]. As a result, the image quality of LSFM is not uniform across the field of view (FOV), often resulting in less-than-ideal datasets. Figure 1A illustrates the problem: a classical light-sheet beam is tightly focused on the center, resulting in clear and sharp images. However, due to diffraction, the edges of the specimen are illuminated by a broader beam, leading to noisy images with reduced axial resolution.

To mitigate the issue of nonuniform image quality in LSFM, researchers seek methods to generate nondiffracting and pseudonondiffracting beams that maintain a consistent illumination profile across the FOV while confining the illumination profile to a narrow plane. Past studies have explored different illumination patterns such as Bessel [36,37] and Airy beams [38,39], which were then combined with LSFM to improve image contrast and resolution. However, debate continues about the ideal illumination beam since quantitative comparisons are challenging due to differences in input pupil structures and NA requirements [35,40].

Here, we showcase an alternative conceptual perspective on the problem by engineering the LSFM illumination beam using DD. DD operates within 2 core layers (Fig. 1B): (a) A physical modeling layer that functions as a simulator, generating the kind of 2D image that an LSFM would capture if confronted with a random distribution of fluorescence emitters within a defined volume and a phase mask delineating the illumination pattern within the volume. (b) A deep learning layer containing a deep learning model that acquires knowledge of the shape of the illumination pattern and detects objects. From simulations, our DD generated a novel LSFM illumination beam that enhanced optical sectioning and which we hereafter refer to as the “butterfly beam” (because the phase mask resembles a butterfly). This innovative illumination beam is the outcome of optimizing hundreds of thousands of variables within the phase mask. Subsequently, we conducted experimental validation, revealing that the butterfly beam exhibits considerably narrower edges within the FOV compared to a Gaussian beam with an identical spatial light modulator (SLM) aperture size.

## Methods

The premise of the study was that enhanced optical sectioning in LSFM leads to better object detection. This assumption dictated our optimization target in simulation, i.e., enhancing the detection of fluorescent emitters within the focal plane while effectively suppressing signals originating from out-of-focus regions. The architecture of our end-to-end network was structured around 2 key components. First, a differentiable optical simulation layer was incorporated, featuring a trainable phase-only mask positioned directly against the illumination objective (Fig. 1A). This component can change the shape of the illumination beam, affording a unique opportunity to optimize the shape of the light sheet for enhanced image quality. The second pivotal component was a convolutional neural network (CNN), renowned for its prowess in feature extraction and pattern recognition. Within our framework, this CNN played an indispensable role by predicting the precise locations of in-focus signals within the simulated images. In this section, we provide the physical and mathematical framework of the problem.

### Optical simulation layer

The optical simulation layer functioned as follows: (a) Initially, we created a random distribution of densely packed beads (3 μm in diameter) within a 3D volume measuring 200 × 200 × 30 μm^3^. (b) We then simulated the light intensity that interacted with each bead within the volume. This 3D intensity distribution changed according to the phase mask pattern. It is important to note that due to the nonideal nature of the generated light sheet, beads located outside the focal plane of the detection objective were also illuminated. Subsequently, we generated the recorded image by considering the noise produced by out-of-focus beads.

#### Simulating the intensity of the variable illumination beam across the 3D volume

To simulate the illumination conditions [41], we first modeled the intensity emitted from the laser as a Gaussian beam defined as:

(1) $$I(x,y)=Ce^{-\left(x^{2}+y^{2}\right)/2w^{2}},$$

where x and y represent spatial coordinates, w is the waist of the input beam, and C is an arbitrary constant. To simplify our simulation, we assumed that the field of the input beam was against the imaging lens. The result was then focused on the multiplied by a defined phase pattern B(x,y) and placed directly sample. The complex field at the focal plane of the illumination lens can be defined as:

(2) $$U\left(u,v\right)=I\left(ℬ\left(x,y\right)\cdot \sqrt{I\left(x,y\right)}\right),$$

where I is the Fourier transform, u and v are the spatial coordinates in the focal plane, defined as $u=f_{x}\lambda F$ and $v=f_{y}\lambda F$, where λ is the illumination wavelength, $f_{x}$ and $f_{y}$ are the spatial frequencies of the Fourier transform, and F is the focal distance of the illumination lens. Next, to propagate the field along the volume of excitation for an arbitrary distance of L, we used the angular spectrum method of propagation. The complex field was defined as:

(3) $$U(u,v,F+L)=I^{-1}\left(I(U(u,v))\cdot H\left(f_{x},f_{y},L\right)\right),$$

where H is the transfer function of propagation through free space:

(4) $$H\left(f_{x},f_{y},z\right)=\left\{\begin{array}{ll} & e^{\frac{2\pi jz}{\lambda }\sqrt{1-{\left(\lambda f_{x}\right)}^{2}-{\left(\lambda f_{y}\right)}^{2}}}\\ 0, & \text{otherwise} \end{array},\;\sqrt{f_{x}^{2}+f_{y}^{2}}<\frac{1}{\lambda }\right..$$

After generating the spatial profile of the beam, the beam was computationally dithered up and down to form the light sheet by averaging the beam in the direction of scan. Note that “dithering” refers to the process of moving the beam up and down by adjusting the angle of the galvo mirror. The entire variable phase mask that determined the optical property of process was modeled in our DD approach, and B(x,y) was the variable phase mask that determined the optical property of the generated beam. B(x,y) was jointly optimized with the detection network.

#### Synthetic image formation

Once the intensity that impinged on each bead in the volume was known, its effect on the detected image needed to be determined. To this end, we calculated the bead point spread function (PSF) as a function of its distance from the detection objective focal plane (z distance) [42]. The defocused incoherent PSF was defined as follows:

(5) $$\begin{array}{l} h(x,y,z)=\\ {\left|C\int_{0}^{1}\;\;J_{0}\left(k\frac{NA}{n}\sqrt{x^{2}+y^{2}}\rho \right)exp\left(-\frac{1}{2}jk\rho^{2}z{\left(\frac{NA}{n}\right)}^{2}\right)\rho d\rho \right|}^{2}, \end{array}$$

where $J_{0}$ is the Bessel function of the first kind, n=1 is the refractive index, C=1 is an arbitrary normalization constant, NA=0.6 is the numerical aperture, λ=561 nm is the detection wavelength, and k=2π/λ is the wave number.

To create a complete synthetic image, we aggregated the individual bead images produced by variable PSFs and combined them with the light-sheet intensity. The result was a synthesized image that provided a comprehensive representation of the observed scene. To ensure the fidelity and realism of our synthesized image, we introduced Poisson noise into the image data. This noise accounted for various factors, including the offset of the image sensor and random background noise. This step was crucial for simulating real-world conditions and improving the accuracy of our results. In Fig. 1B, a physical model is presented to illustrate the process of generating a synthetic image.

### Joint optimization of the phase mask and object detection network

To detect the locations of the beads, we used a rudimentary CNN that contained only 6 ResNet layers with shortcut connections. State-of-the-art object detection networks did not fit into our limited graphics processing unit (GPU) memory (32 GB) given the incorporation of the complex optical simulation layer. The end-to-end deep learning pipeline is illustrated in Fig. 1B. The physical model took the random bead locations (x,y,z), and given a phase mask, it generated synthetic images (pixel size 1 × 1 μm^2^). The network component consisted of repeated convolutional layers with a kernel size of 3 × 3, followed by a leaky ReLU activation function and batch normalization. Max pooling was applied to perform downsampling to reduce computational cost. The output of the network was a 2D grid with a pixel size of 4 × 4 μm^2^ that indicated the position of in-focus beads. The detailed network structure is shown in Fig. S1.

Because the optical model was differentiable, the network parameters and phase masks could be jointly optimized simultaneously. The joint network was implemented in Python 3.7 with PyTorch-1.12.0 Deep Learning Framework and trained on a single Nvidia Tesla V100–32GB GPU on the UNC Longleaf cluster for approximately 72 h. The prediction of in-focus beads was treated as a classification problem, and the binary cross entropy with logits loss function was used. Classifying in-focus beads enabled us to filter out beads that were out of focus or exhibited a low signal-to-noise ratio, ensuring that only the in-focus beads were detected. The loss function was calculated between the ground truth of the in-focus 2D grid of beads and the output grid of the network. In this approach, we treated the output as a pixel-wise binary classification task. Each pixel in the grid was classified as either belonging to an in-focus bead (in which case the value is 1) or the background (in which case the value is 0). The binary cross entropy loss function works by penalizing the predicted probabilities based on the ground truth in-focus 2D grid. Additionally, monitoring metrics such as accuracy, precision, recall, and F1 score during training and validation can provide a more holistic view of our joint optimization model. The learning rate was set to 0.01 with the Adam optimizer. The pixel size of the phase mask and synthetic image was 1 × 1 μm^2^, and the sizes of the phase mask and synthetic image were set to 500 × 500 μm^2^ and 200 × 200 μm^2^, respectively. Simulated beads were randomly distributed in the 200 × 200 × 30 μm^3^ space. Details about the parameter settings can be found in the source code.

### Custom-built light-sheet design

LSFM was inspired by previous designs [43–46], and the basic setup and complete list of components can be found in our recent studies [30,44]. Here, we duplicated the setup and added an SLM to reshape the illumination beam (see Fig. S2). Briefly, a Gaussian beam emitted from a continuous-wave laser (Coherent; OBIS LS 561–50) served as our illumination source. The beam passed through a half-wave plate (Thorlabs; WPH10M-561) that was mounted on a rotating fixture. We expanded the beam and used a scanning galvo system (Cambridge Technology; 6215H) to introduce dithering up and down the illumination beam, which consequently generated the light sheet. This scanning galvo system was conjugated onto an SLM (Medowlark; E19X12 series) to optimize the phase within the beam path. Conjugation between the galvo and SLM was achieved using a 4f optical system. The first lens of this system, a scan lens (Thorlabs; CLS-SL-70 mm), and a second lens, a refractive lens (Thorlabs; AC508–150-A-ML), were employed in the setup. The beam, now modulated by the SLM, underwent focusing through a lens (Thorlabs; AC508–100-A-ML), with a slit positioned at the front focal plane of the lens. The purpose of the slit was to eliminate the zeroth-order (unmodulated) light while collecting only the modulated light. Further demagnification was achieved through a combination of a 2× lens (Keyence; BZ-PF10P 0.1 NA) and a 10× lens (Olympus; RMS10X-PF 0.30 NA), resulting in a slenderer beam. Subsequently, this refined beam was projected onto a cuvette (Thorlabs; CV10Q35FAE) filled with Dibenzyl ether serving as the chamber for imaging tissue-cleared samples. The interface between the lens and cuvette was filled with immersion oil to minimize aberrations. Additionally, to correct for any illumination aberrations, the Gaussian beam aberrations were recorded and corrected using the SLM, and the corrections were used as constant biases on the SLM pattern. The placement of the sample within the cuvette was facilitated by a customized ASI-3D stage, allowing for precise 3D adjustments during imaging. To capture the fluorescent signal, a 10× objective lens (Mitutoyo; Plan Apo 0.28 NA) was employed, and an emission filter (AVRO; FF01–593/40–25) was inserted into the detection path to eliminate unwanted signals. The detected signal was then collected by a tube lens (ASI; TL180-MMC), followed by a complementary metal-oxide-semiconductor (CMOS) camera (Hamamatsu; C13440–20CU).

In addition to the imaging wing, a beam characterization wing was also added along the optical axis of the propagating beam in which a 10× lens (Olympus; RMS10X-PF 0.30 NA) microscope objective was mounted on a translational stage (Newport; 561D-XYZ and CONEX-TRB12CC motor), and the beam profile was captured using a combination of a tube lens (ASI; TL180-MMC) and a CMOS camera (Hamamatsu; C13440–20CU). A graphical user interface written in MATLAB (2019b) was used during image acquisition.

### Sample preparation

Two mouse brains [47] and 4 prairie vole brains were subjected to pretreatment involving methanol, immunolabeling, and clearing [48]. Postnatal day 30 mouse brain samples were stained using chicken anti-green-fluorescent protein (GFP with Alexa Fluor 647 as the secondary antibody) and rabbit anti-red-fluorescent protein (RFP with Cy3 as the secondary antibody). Postnatal day 60 vole brains were stained using mouse anti-oxytocin (with Alexa Fluor 647 as the secondary antibody) and rabbit anti-vasopressin (with Alexa Fluor 555 as the secondary antibody). The harvesting of all animals was conducted in accordance with the regulations and approval of the Institutional Animal Care and Use Committee at North Carolina State University.

## Results

In this section, we present the results of our simulations, experiments, and LSFM imaging. As detailed below, the simulation results show that the butterfly beam improves object detection; the experimental results show that the realized butterfly beam shape and profile are similar to the simulated beam shape and profile; and the LSFM imaging results show that the butterfly beam improves axial resolution at the edge of the FOV in comparison with the Gaussian beam.

### Simulation results

**Fig. 2.**
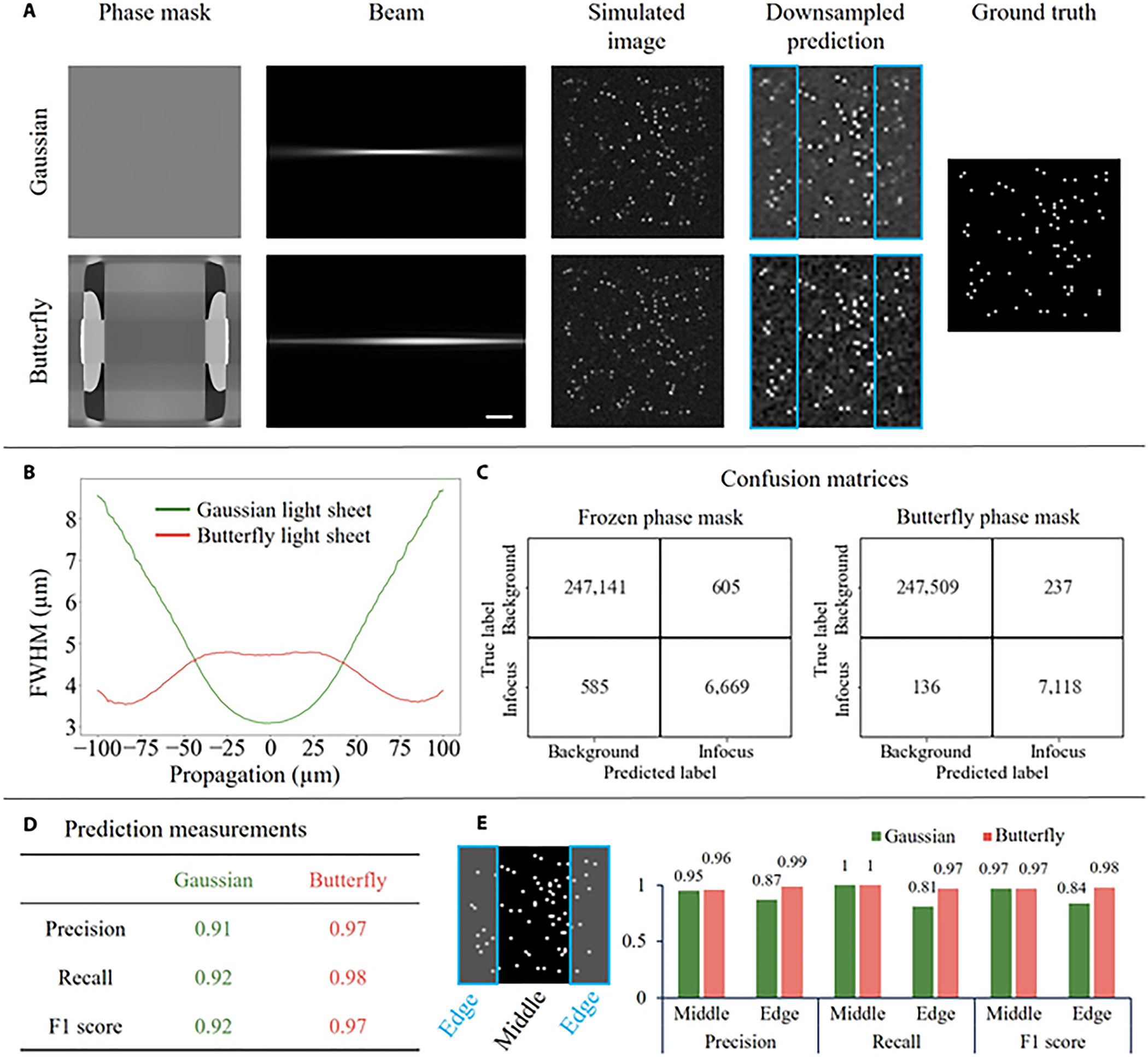
Simulation of DD-mediated optimization. (A) Optimized phase mask to improve bead detection accuracy. Compared with a Gaussian beam (flat phase mask), the butterfly beam exhibits better image contrast on the edges (see inside the blue rectangular areas), rendering a better prediction. (B) Beam profile comparison. FWHM for Gaussian and butterfly beams plotted against the direction of propagation. The graph illustrates that the butterfly beam exhibits reduced FWHM at the edges, while the Gaussian beam is narrower at the center of the field of view. (C and D) Network performance. A comparison of classification metrics (confusion matrix and prediction measurement) between frozen and butterfly phase scenarios. The optimized phase mask demonstrates improved bead detection capability and achieves higher scores across the evaluated metrics. (E) Comparison between middle and edge areas. The field of view is divided into middle and edge areas. The metrics are calculated separately, and the butterfly beam provides better performance on the areas near the edges.

We trained our end-to-end deep learning model on randomly distributed beads in 3D space, starting with an initial phase mask of all zeros, which generated a conventional Gaussian light sheet. Through each iteration, the phase mask was updated together with the object detection network to improve detection accuracy. In the simulation, the DD method produced a phase mask that resulted in a light sheet with less diffraction along the propagation axis (Fig. 2A) after 50 epochs. Figure 2B illustrates the calculated full width at half maximum (FWHM) of the butterfly (optimized) and Gaussian beams. The FWHM was determined by collapsing the beam along the dithering direction. A Gaussian fit was then applied to the averaged beam, and the FWHM was calculated based on the standard deviation (SD) as FWHM = 2.355 × SD. The butterfly beam shows a much narrower width at the edges of the FOV, while slightly sacrificing the center of the FOV. The FWHM increase in the center of the FOV can be attributed to several factors. First, a trade-off exists between the center and the edges as the algorithm strives to improve sectioning across the entire FOV. Second, in this specific scenario, the DD approach may have converged to a local minimum instead of a global one. To address these challenges, various strategies can be implemented: (a) experimenting with different optimization algorithms, such as stochastic gradient descent and root mean square propagation, instead of the Adam optimizer; (b) implementing learning rate scheduling techniques; and (c) utilizing regularization techniques, such as modifying the network structure or applying L1/L2 regularization to network parameters. A detailed cross-section of the Gaussian and our butterfly beam in simulation is provided in Fig. S3. Simulation of the butterfly beam produced cleaner images at the edges (i.e., less background noise) in comparison with the Gaussian beam (Fig. 2A, blue boxes).

The output of the DD approach was an object detection network tailored to the butterfly beam illumination profile. Therefore, we validated that the improvement in image quality at the edges of the FOV was translated to improvement in the downstream analysis (Fig. 2C to E). For an unbiased comparison, our baseline\gold standard network was trained from scratch on images that were produced solely by a Gaussian beam. We refer to this network as the “frozen phase mask” network since it was not updated from a uniform phase during the training. Figure 2C shows the confusion matrix for 2 classes: background and in-focus beads. The overall F1 scores (indicating class-wise performance) of the butterfly and Gaussian beams were 0.97 and 0.92, respectively (Fig. 2D). Since the butterfly beam was narrower at the edges of the FOV, we also compared the object detection results between the center and the edges of the FOV (Fig. 2E). We expected that the object detection in conjunction with the butterfly beam would outperform the Gaussian beam on the edges, and this expectation was confirmed by the simulation. For example, at the edge of the FOV, the F1 scores of the butterfly and Gaussian beams were 0.98 and 0.84, respectively. To evaluate the robustness of DD, we ran DD under different noise levels and initial guess conditions. “Initial guess conditions” refers to the initialization values of the phase mask when initiating DD optimization. Remarkably, in all tested cases, the butterfly beam pattern emerged as the most optimal illumination beam (Figs. S4 and S5). As an additional quality check for our DD approach, we also tested it in perturbation experiments (Fig. S6). For example, when we focused the beam waist away from the center of the FOV, the network repositioned it by producing a lens-like pattern (Fig. S6). Qualitatively, the same butterfly beam shape was observed in these perturbation experiments.

### Experimental results

**Fig. 3.**
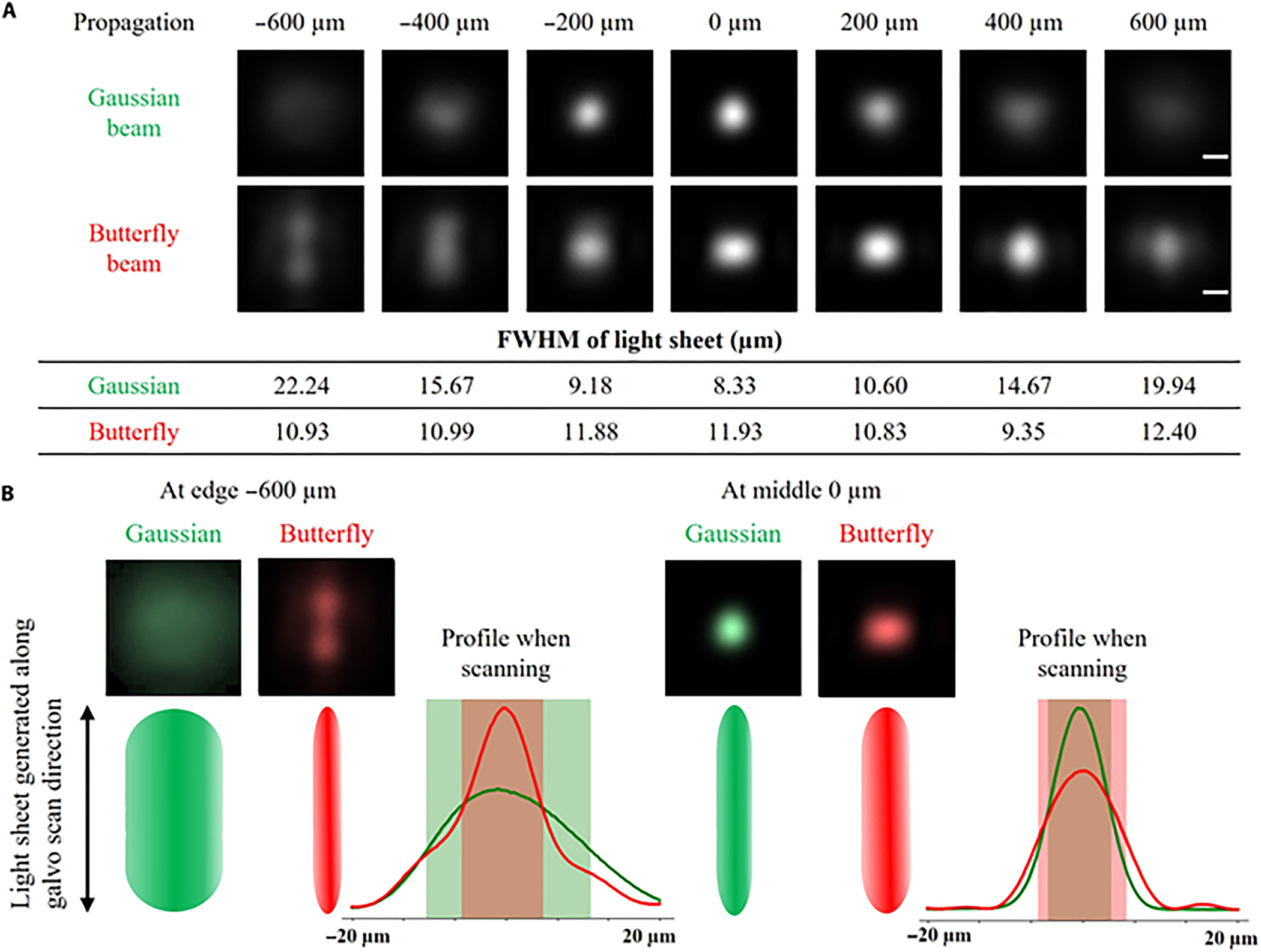
Experimental results—characterization of butterfly beam. (A) Static profiles of Gaussian and butterfly beams along beam propagation. The propagation distance of 0 μm corresponds to the focal point of the excitation objective, where the Gaussian beam reaches its minimal waist. As the light sheet is generated by dithering the static beam up and down, light-sheet thickness was measured by summing the values along the columns. Experimental measurements of the beam were conducted using a single lens after the spatial light modulator, outside the immersion chamber. The scale bar is 10 μm. (B) Schematic depicting formation of light sheet through up-and-down dithering of static beam. The line profile along the scanning direction compares the Gaussian beam (in green) with the optimized/butterfly beam (in red). To maintain a narrow profile at the edges of the field of view, the deep design optimization elongated the profile in the direction of the scan.

Next, we experimentally tested the properties of the butterfly beam. Figure 3A reveals, consistent with the simulation, that the experimentally measured FWHM of the butterfly beam was wider at the center, measuring 11.93 μm, compared to both edges of the FOV, for example, 9.35 μm (at 400 μm) and 10.93 μm (at −600 μm). This contrasted sharply with the Gaussian beam (featuring a constant phase mask on the SLM), where the FWHM was narrower at the center, measuring 8.33 μm, compared to both FOV edges, for example, 14.67 μm (at 400 μm) and 22.24 μm (at −600 μm). Conceptually, these numerical values closely paralleled our expectations (Fig. 2).

Note that the experimental setup underwent modifications from the simulation. This adjustment was necessitated by the need to utilize very short distances during the simulation to replicate high-NA conditions within the constraints of limited GPU memory. Within these brief distances (e.g., 2 mm), the integration of optical components in an experimental setup proved unfeasible. Consequently, the primary disparities between the simulation and experimental setup were as follows: (a) The physical size of the SLM exceeded the dimensions of the phase mask in the simulation. (b) For the generation of the butterfly beam, the SLM was positioned in the back focal plane of a 1″ lens with a 125-mm focal distance (Thorlabs; AC254–125-A-ML). (c) The SLM featured constant phase patterns for aberration compensation and a linear phase mask to reject the zeroth order. The beam profiling process involved imaging the beam using a 10× objective (Olympus RMS10X-PF 0.30 NA), which was mounted on a translation stage and positioned at the focal plane of the illumination lens in air. To capture the beam profile at different propagation distances, the microscope objective was translated to various locations. At each location, the beam was imaged using a joint system comprising a tube lens and camera. This methodology facilitated the comprehensive characterization of the beam profile across different propagation distances.

Figure 3B illustrates the utilization of static beams for generating the light sheet. These beams underwent dithering up and down, meaning that the light sheet was formed by averaging the profile in the direction of the scan. Notably, the DD optimization disrupted the symmetry of the beam and elongated it in the direction of the scan, a phenomenon that does not impact the FWHM. This optimization demonstrates a sophisticated approach and a physical explanation of the obtained butterfly beam profile.

### LSFM imaging results

Next, we imaged samples using the butterfly beam and the setup described in the “Custom-built light-sheet design” section. Again, we resized the butterfly phase mask to match the dimensions of the SLM and we multiplied the phase pattern by a constant. The resizing was carried out to optimize the achievable NA for the system, while the multiplication factor accommodated variations arising from the increasing complexity of the imaging system and the scaling of the phase mask. We empirically found that a multiplication factor of 160 demonstrated the most considerable improvement in the beam profile (Fig. S7). The empirical estimation was performed by capturing the beam profile at various axial locations along the direction of beam propagation within the LSFM system, considering the different multiplication factors (Fig. S2, beam characterization wing). During the beam characterization mode of the LSFM system, the chamber was filled with Dibenzyl ether and no sample was inserted into it. This multiplication factor was imaging system dependent.

**Fig. 4.**
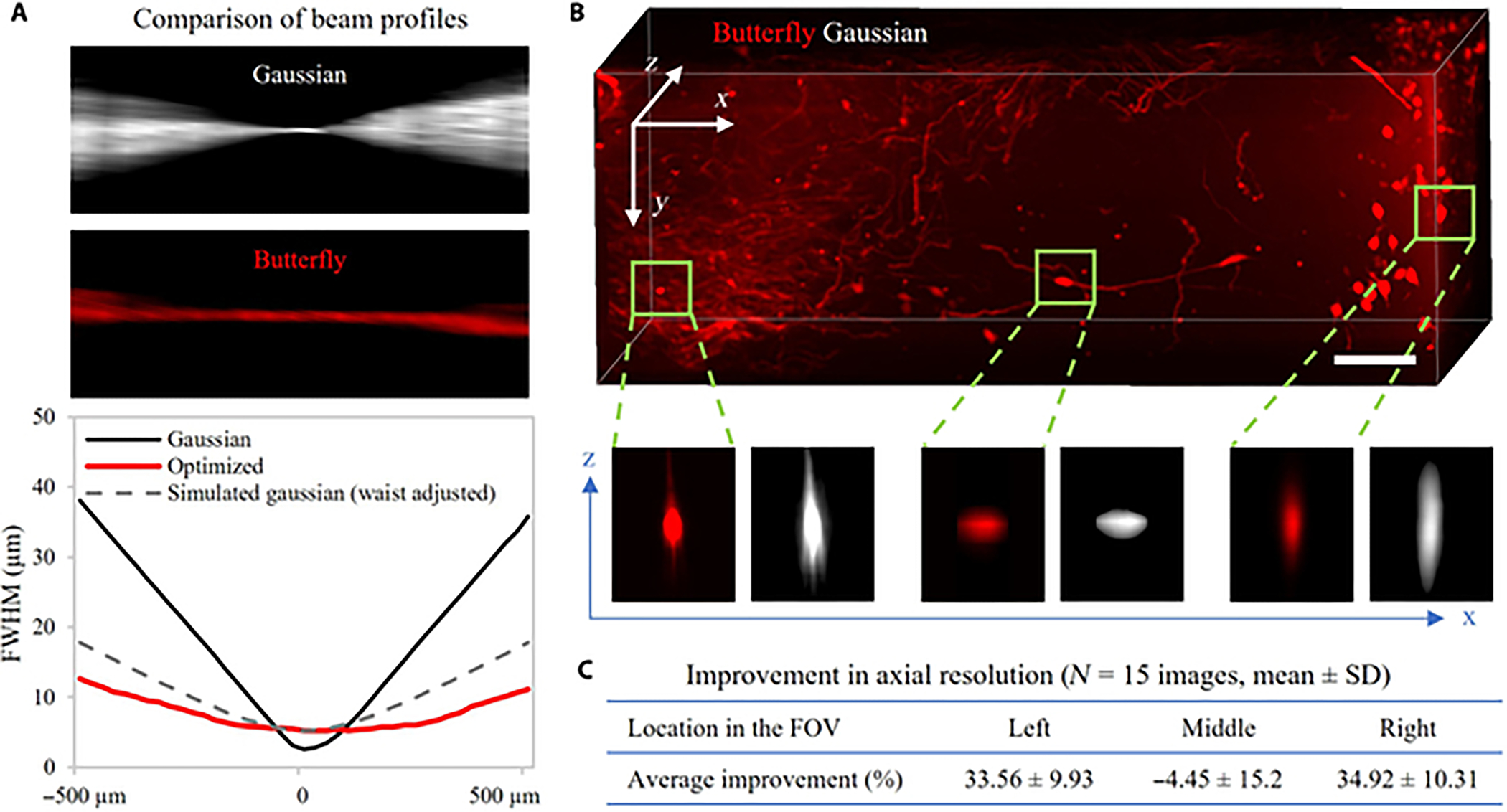
Experimental results for LSFM-based imaging. (A) Comparison of beam profiles. The beam profile and the FWHM curve demonstrate that the deep design “butterfly” phase mask provides narrower illumination at the edges of the FOV. Note that the butterfly beam has a narrower profile at the edges, even when simulating a Gaussian beam with a similar waist. (B) Maximum intensity projection image of z-stack acquired from tissue-cleared mouse brain. The zoomed-in images demonstrate that the butterfly beam (red) exhibits a superior axial point spread function (XZ profile) compared to the Gaussian beam, particularly at the edges. The scale bar is 100 μm. (C) Improvement in axial resolution. The butterfly beam has a narrower axial profile in the edges of the FOV, whereas in the middle, the axial profile is comparable to that of the Gaussian beam.

Figure 4A illustrates the beam profiles in the direction of propagation for both the Gaussian and butterfly beams across the FOV (Fig. S2, beam characterization wing). Additionally, Fig. 4A displays the beam waist for the butterfly beam compared to the Gaussian beam at different positions across the FOV. It also provides a comparison with a simulated Gaussian beam, assuming its beam waist matches that of the butterfly beam at the focal plane. This comparison underscores that even when the butterfly beam possesses a large beam waist, it outperforms a Gaussian beam of the same beam waist.

In the next series of experiments, we imaged tissue-cleared mouse brain samples in which neurons were sparsely labeled with genetic fluorescent reporters. Figure 4B showcases a 3D volume of a brain sample, with the image obtained using the butterfly beam (red; see Movie S1) and the Gaussian beam (white; see Movie S2). The axial profiles of several neurons revealed that in the middle region of the FOV (i.e., where the beam waists are similar), the Gaussian and butterfly beams perform similarly (Fig. 4B, lower half, and Fig. S8). However, as we move to the edges of the FOV (i.e., where the butterfly beam exhibits a narrower profile), it becomes evident that the axial resolution of the butterfly beam surpasses that of the Gaussian beam. We conducted a comparison of the FWHM in the axial axis for both beams across 15 randomly selected neurons in each location in the FOV (Fig. 4C) obtained from 2 brains. The butterfly beam delivered superior optical sectioning at the edges both qualitatively and quantitatively (33% to 35% improvement in both edges). The Gaussian beam provided slightly (approximately 4.5%) superior results to the butterfly beam in middle, in line with our earlier simulation results.

## Discussion

LSFM has emerged as the preferred method for high-throughput imaging, generating extensive imaging datasets that are commonly analyzed using deep learning models. With the data analysis stage becoming a bottleneck in utilizing LSFM, there is growing demand for approaches that strategically optimize the data acquisition stage to enhance the downstream analysis. Several pioneering reports have supported this transformative approach. For instance, deep-STORM was used to enhance super-resolution single-molecule microscopy, and “learned sensing” was used to improve the detection of malaria-infected cells [20,22]. Nevertheless, existing methodologies employ comparatively basic optical models that do not include the intricate propagation of waves within a volume.

In contrast, our approach leverages an optical model that considers the wave propagation of the illumination beam within the excitation volume. Through the utilization of this model, we jointly fine-tuned an object detection network and a phase mask, collectively shaping the illumination beam for improvement in object detection. This approach is supported by the capability of the SLM to dynamically modulate the illumination beam, and the ability to simulate the SLM as a large filter in the network structure. Our DD approach involved devising the butterfly beam using simulation, which we experimentally validated, revealing that the butterfly beam exhibits considerably narrower edges within the FOV compared to a Gaussian beam with an identical SLM aperture size. With the narrower beam at the FOV periphery and an objective with an ample depth of field (0.28 NA), we anticipated an immediate enhancement in axial resolution when employing the butterfly beam, as opposed to the Gaussian beam. This observation was consistently validated across tissue-cleared brain samples, with the butterfly beam yielding a 33% to 35% improvement in axial resolution compared to the Gaussian beam.

Upon retrospective examination of the shape of the butterfly beam, a logical explanation emerges. The generation of the light sheet involves dithering the static profile of the beam up and down (Fig. 3B). Our DD approach capitalizes on this phenomenon, resulting in an asymmetrically elongated butterfly beam along the direction of the scan. Meanwhile, it ensures that the FWHM of the perpendicular axis remains narrow (Fig. 3B). Notably, any elongation or aberrations in the direction of the scan of the static beam fail to impact image quality, as the beam is scanned in this direction. The perpendicular axis (thickness of the light sheet) governs the optical sectioning in LSFM, which the butterfly beam safeguards by maintaining its narrow profile. Upon examination, this approach proves to be physically sound, achieved through pure optimization.

While other illumination beams, such as Bessel and Airy beams, may yield superior axial resolution compared to the Gaussian beam, they essentially balance axial resolution with contrast due to their considerable sidelobes. The butterfly beam, with its mild sidelobes, does not degrade the contrast as extensively. Furthermore, the DD approach can be customized for any application and optimization objectives, provided they can be defined mathematically. For example, DD can be fine-tuned for variable emitter concentrations within a volume or optimized for different loss functions, including increased contrast or resolution. Furthermore, the DD approach presents clear advantages over image enhancement algorithms (postacquisition enhancement). Unlike such algorithms, which generate intermediary results requiring separate deep learning analysis for information extraction, DD is crafted for end-to-end optimization. The inclusion of image-enhancement algorithms adds an extra layer to the analysis process with no assurance of improving downstream results, since information theory suggests that postprocessing alone fails to augment information [49]. In contrast, our DD approach is specifically designed for optimizing the acquisition process to directly enhance information extraction during the image acquisition phase.

An obvious limitation of our present results is that we do not use the jointly optimized deep learning model to further enhance the image quality, and we only report the raw images that were captured using the butterfly beam. The reason is that the jointly optimized deep learning model (blue pyramid in Fig. 1B) is trained on downsampled images, and therefore applying it will result in severely degraded resolutions. We did not train our companion network on the high-resolution images since the entire image formation model and a large object detection network could not fit within our limited 32 GB GPU memory. This limitation in GPU memory will be addressed in future studies, given the constant improvement in the availability and price of GPUs with more memory. An additional limitation of our DD method is that the illumination pattern and the network could converge into local minima, and therefore an optimal solution may be unachievable. We partially addressed this issue by extending our simulation datasets and by utilizing stochastic gradient descent methods that help to escape local minima. Furthermore, the selection of the loss function employed can considerably impact both the performance and convergence of the model. Based on our observations, various loss functions such as hinge loss or focal loss can be utilized in future studies.

Overall, the present results constitute an important stride in exploring novel avenues for system optimization using DD, highlighting the immense potential of our approach to elevate performance across not only imaging pipelines, but also other diverse applications. Our results underscore the importance of integrating physical models into the DD framework to advance the performance of optical system design. It constitutes a paradigm shift in the field of optical system design when data are automatically analyzed by deep learning models.

## Supplementary Material

Figs. S1 to S8 and Movies S1 and S2

## Data Availability

The source code of the training end-to-end deep learning model in this paper can be obtained at https://github.com/chenli38/DL_phase_optimization.
