# Supplementary material for: Intelligent Beam Optimization for Light-Sheet Fluorescence Microscopy through Deep Learning
Source: Intell Comput. Author manuscript; Available in PMC 2024 Aug 4. (PMC11298055; doi:10.34133/icomputing.0095)
Supplement: Figs. S1 to S8 and Movies S1 and S2 [file NIHMS2009356-supplement-Figs__S1_to_S8_and_Movies_S1_and_S2.zip › ICOMPUTING-D-24-00003 Supplemental Material.docx]

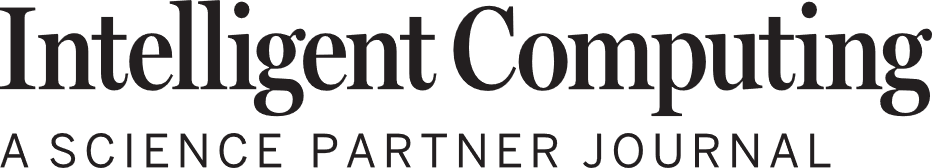


Supplementary Materials for

**Intelligent Beam Optimization for Light-Sheet Fluorescence Microscopy through Deep Learning**

**Chen Li, Mani Ratnam Rai, Yuheng Cai, H. Troy Ghashghaei, and Alon Greenbaum**

Corresponding author: Alon Greenbaum, greenbaum@ncsu.edu

**The PDF file includes:**

Figs. S1 to S8

**Other Supplementary Materials for this manuscript include the following:**

Movies S1 to S2

**Supplementary Figures**


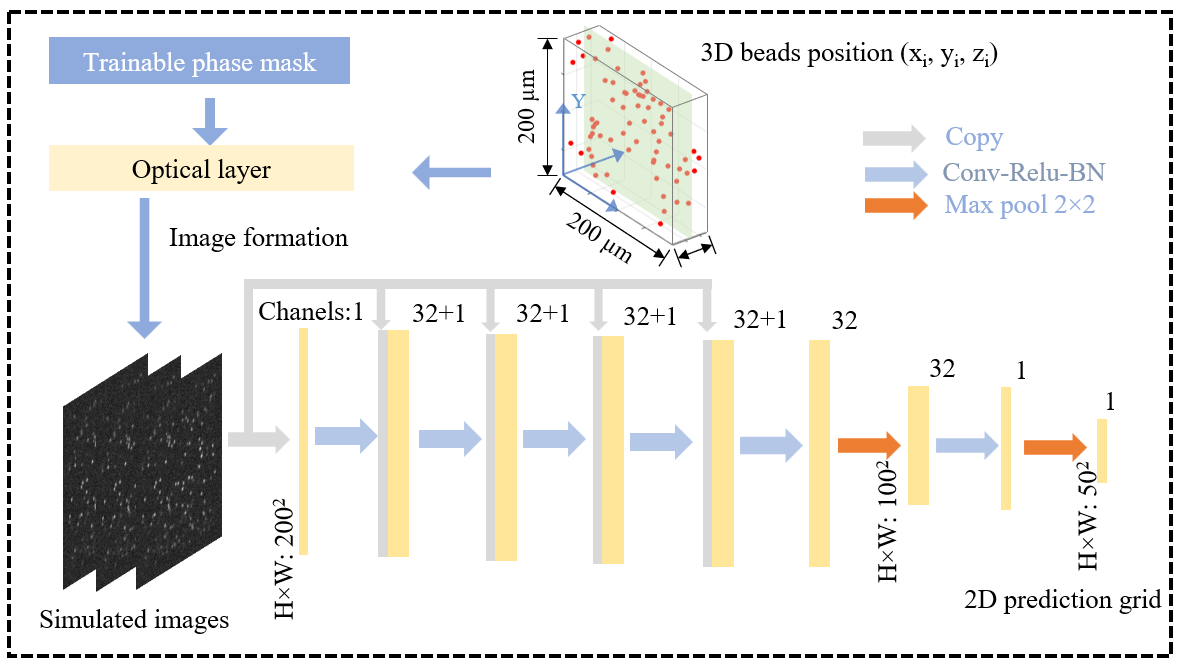


**Fig. S1. Detailed network structure used for training.** H – number of pixels in the horizontal direction, W – number of pixels in the vertical direction. Annotation 32+1 – employing 32 filters and a shortcut connection.


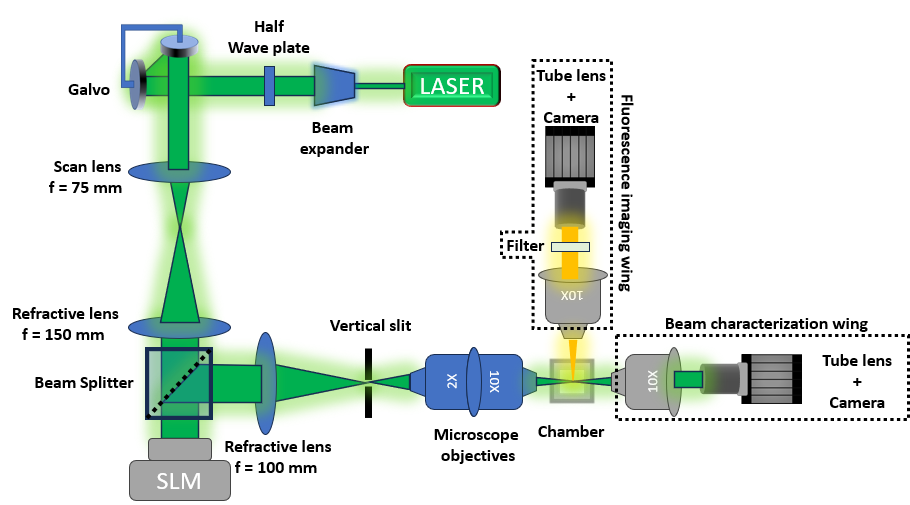


**Fig. S2. Detailed optical design of LSFM system for beam characterization and imaging.** The experimental imaging system comprised 2 wings. The beam characterization wing facilitated the capture of the beam profile along its propagation, while the fluorescence imaging wing was dedicated to capturing the fluorescence signal emitted by the sample. A detailed list of components and their placement can be found in the “Custom-built light-sheet design” section.

*
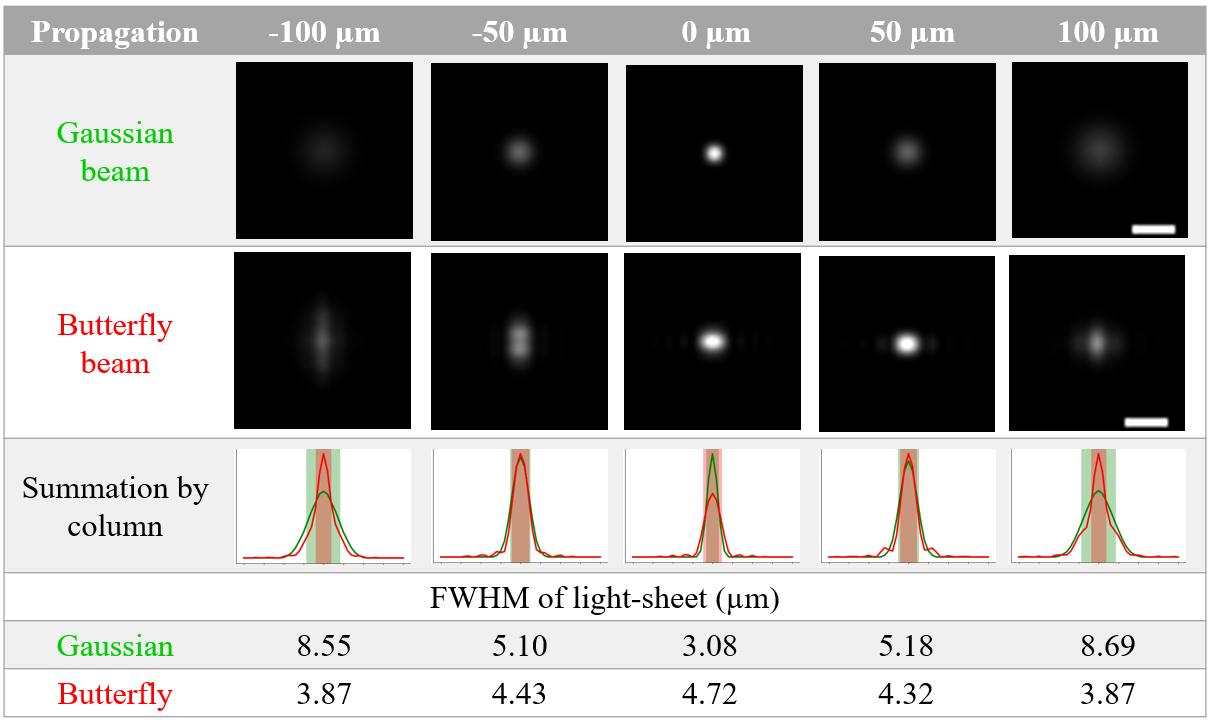
*

**Fig. S3.** **Beam profile analysis in simulation**. In the top panels, images depict the static profiles of Gaussian and optimized/butterfly beams along the beam propagation. The propagation distance of 0 μm corresponds to the focal point of the excitation objective, where the Gaussian beam reaches its minimal waist. As the light sheet is generated by dithering the static beam up and down, light-sheet thickness was measured by summing the values along the column in simulations. Simulation results show that the optimized beam is narrower at the edges of the field of view. The scale bar is 10 µm.

**
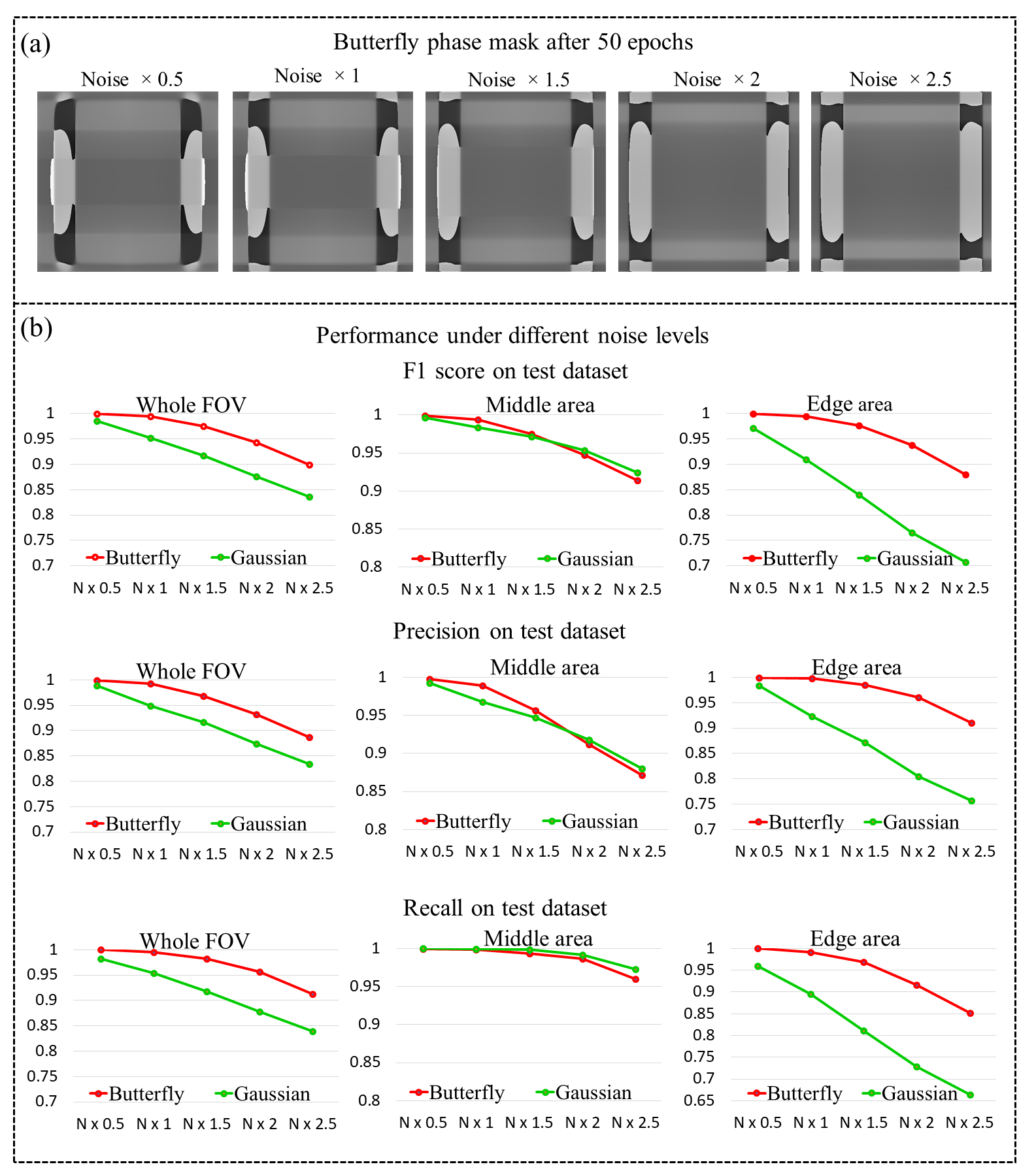
**

**Fig. S4. Deep design convergences under varying noise levels in simulation.** **(a)** **Optimized phase after 50 epochs.** The butterfly phase mask shows consistent patterns despite different levels of Poisson noise, where the standard deviation is multiplied by an increasing factor. This suggests that the butterfly phase pattern generalizes well across varying noise levels. **(b)** **F1 scores, precision, and recall under different noise levels using Gaussian and butterfly beams.** Results computed on 100 test images across the entire, middle, and edge areas of the field of view. The x-axis indicates the increasing noise level, e.g., $N\times2$ represents a simulation with double the noise level of $N\times1$. As anticipated, the butterfly beam performs better than the Gaussian beam at higher noise levels.

**
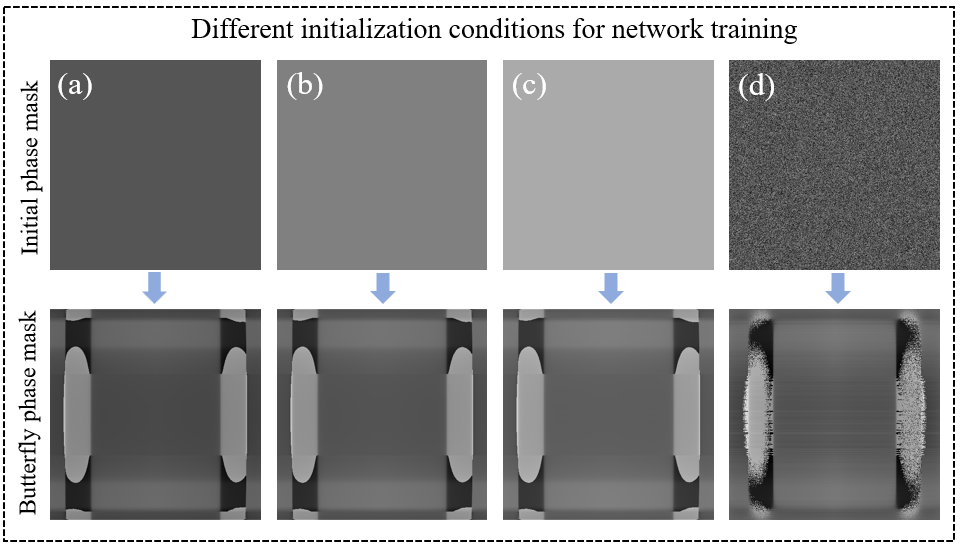
**

**Fig. S5. Convergence of deep design under various initial guesses for phase mask. (a) to (d) Initial guesses for phase mask (0, 0.5, 1, and random noise).** Despite different initial conditions, all 4 instances converge to conceptually similar phase masks—ultimately resembling the butterfly phase mask.

**
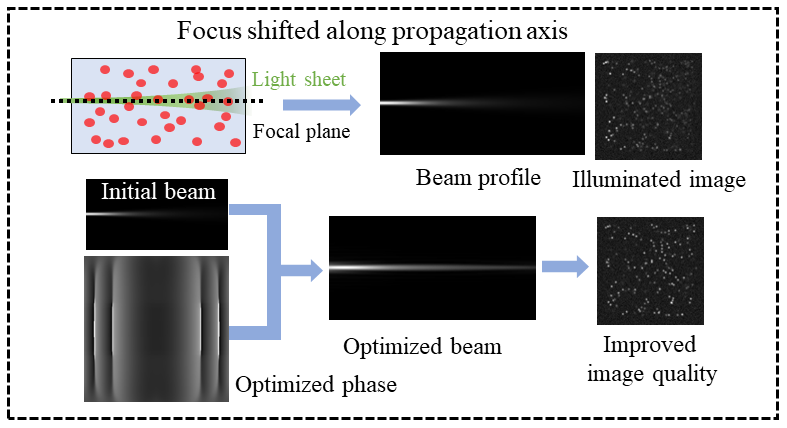
**

**Fig. S6. Perturbation test for DD.** In instances where the simulation consistently introduces errors in the beam profile—such as focusing on the edge of the field of view under a flat phase mask—DD centers the beam. Conceptually, the resulting phase mask incorporates a lens-like pattern atop the butterfly beam. These results underscore the robustness of our approach and its capability to manipulate the light sheet in 3D space.


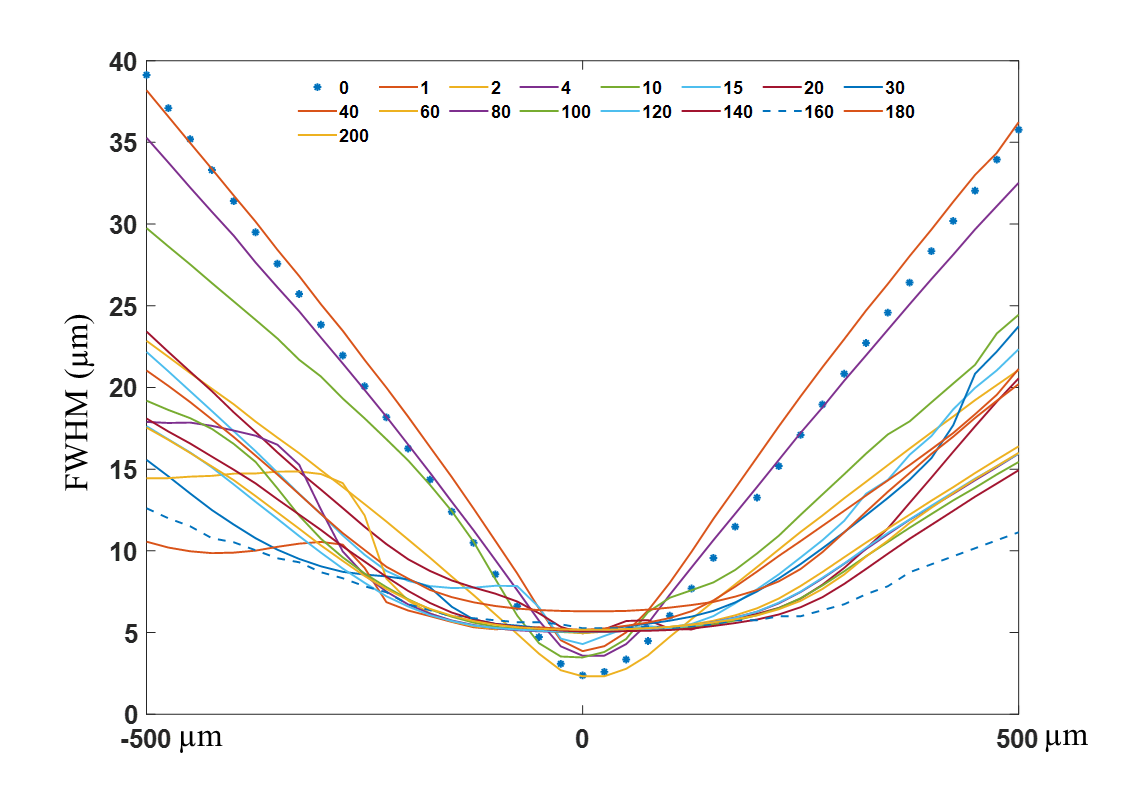


**Fig. S7. Full width half maximum (FWHM) for various modulation factors applied to optimized phase mask.** In the presence of additional optical elements for imaging tissue-cleared samples, the phase mask was multiplied by a modulation factor. These elements encompassed 2 objective lenses and a sample cuvette filled with immersion media. They were omitted from the simulation, but were required for imaging tissue-cleared samples. See Fig. S2 for the beam characterization setup.


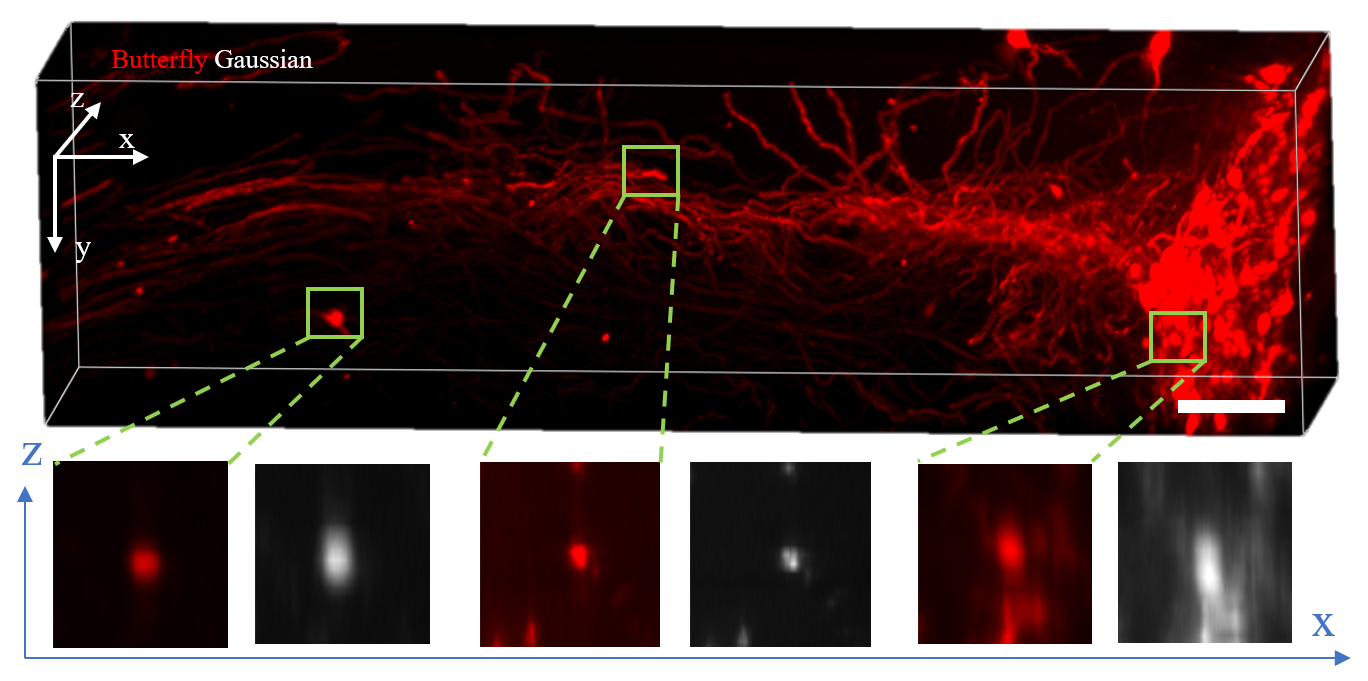


**Fig. S8. Additional experimental results for LSFM-based imaging using butterfly beam**. Maximum intensity projection image of a z-stack acquired from a tissue-cleared mouse brain. The zoomed-in images and axial profile (XZ) clearly demonstrate that the butterfly beam (red) exhibits a superior axial point spread function compared to the Gaussian beam, particularly at the edges of the field of view. The scale bar is 100 µm.

**Not Included in PDF**

Movie S1.
3D reconstruction of the imaged brain using butterfly beam.

Movie S2.
3D reconstruction of the imaged brain using Gaussian
