# Supplementary figures and images for: Intelligent Beam Optimization for Light-Sheet Fluorescence Microscopy through Deep Learning
Source: Intell Comput. Author manuscript; Available in PMC 2024 Aug 4. (PMC11298055; doi:10.34133/icomputing.0095)

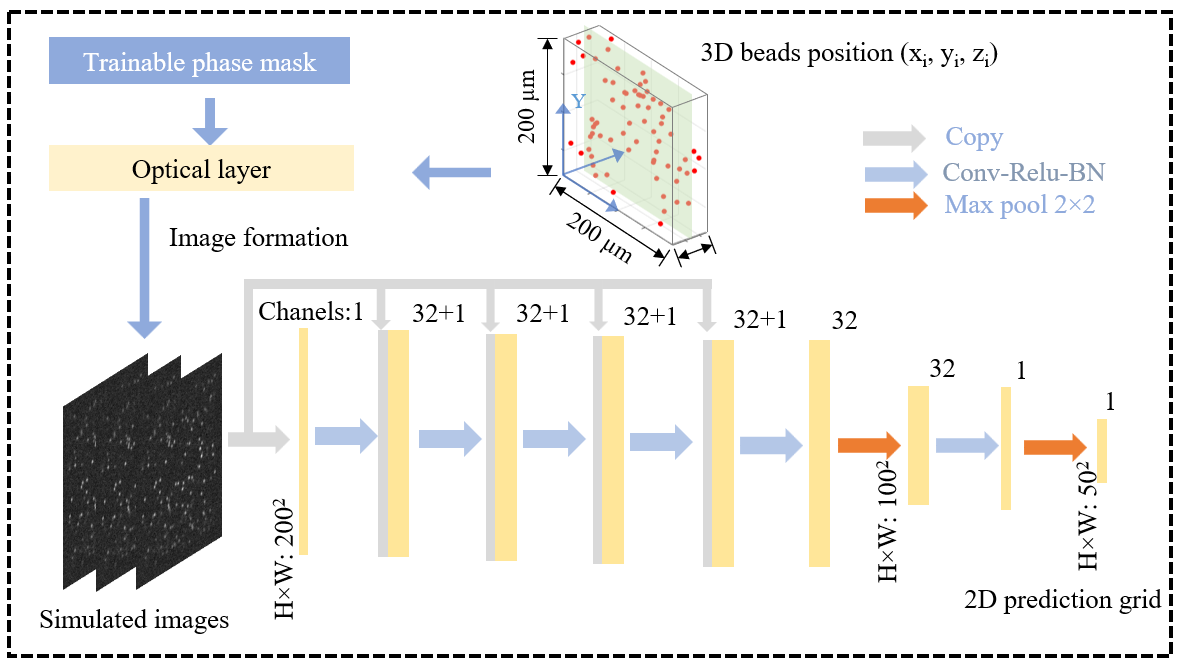

Supplement: Figs. S1 to S8 and Movies S1 and S2 [file NIHMS2009356-supplement-Figs__S1_to_S8_and_Movies_S1_and_S2.zip › S1.png]

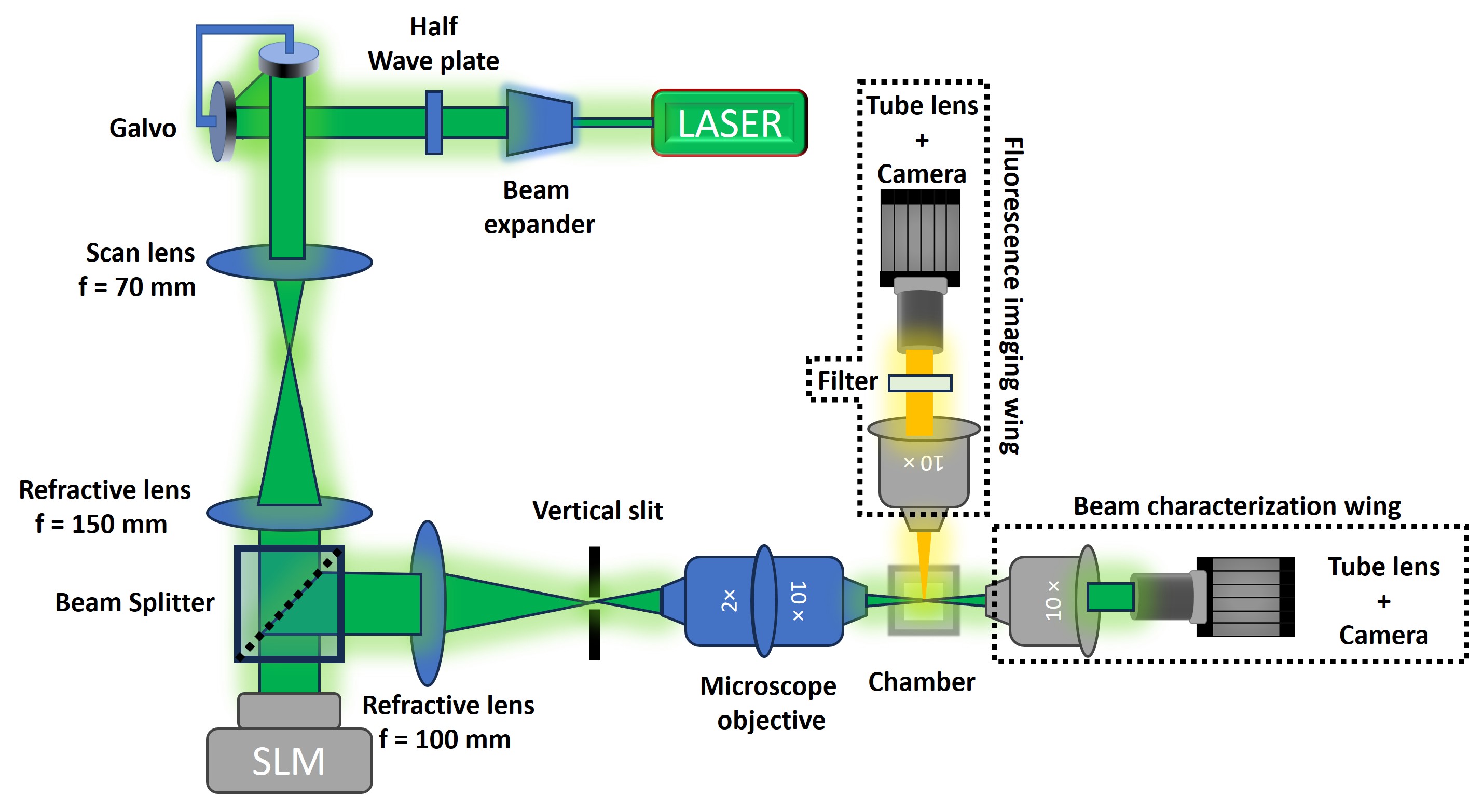

Supplement: Figs. S1 to S8 and Movies S1 and S2 [file NIHMS2009356-supplement-Figs__S1_to_S8_and_Movies_S1_and_S2.zip › S2.jpg]

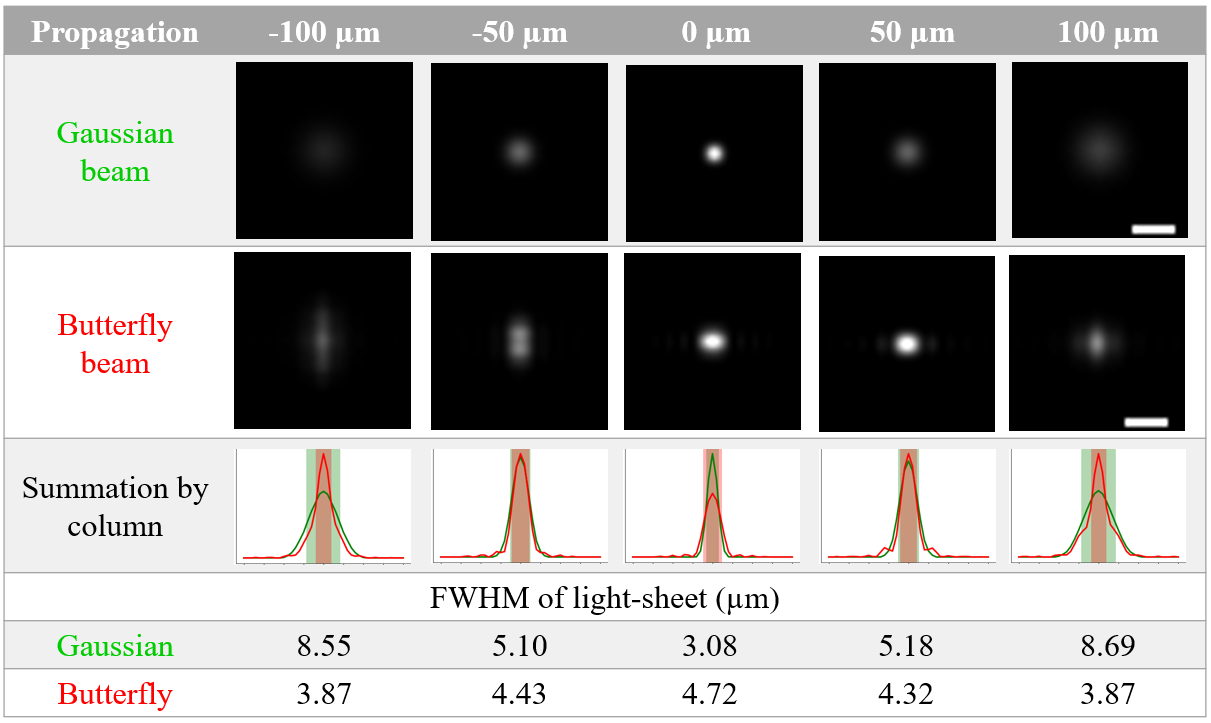

Supplement: Figs. S1 to S8 and Movies S1 and S2 [file NIHMS2009356-supplement-Figs__S1_to_S8_and_Movies_S1_and_S2.zip › S3.png]

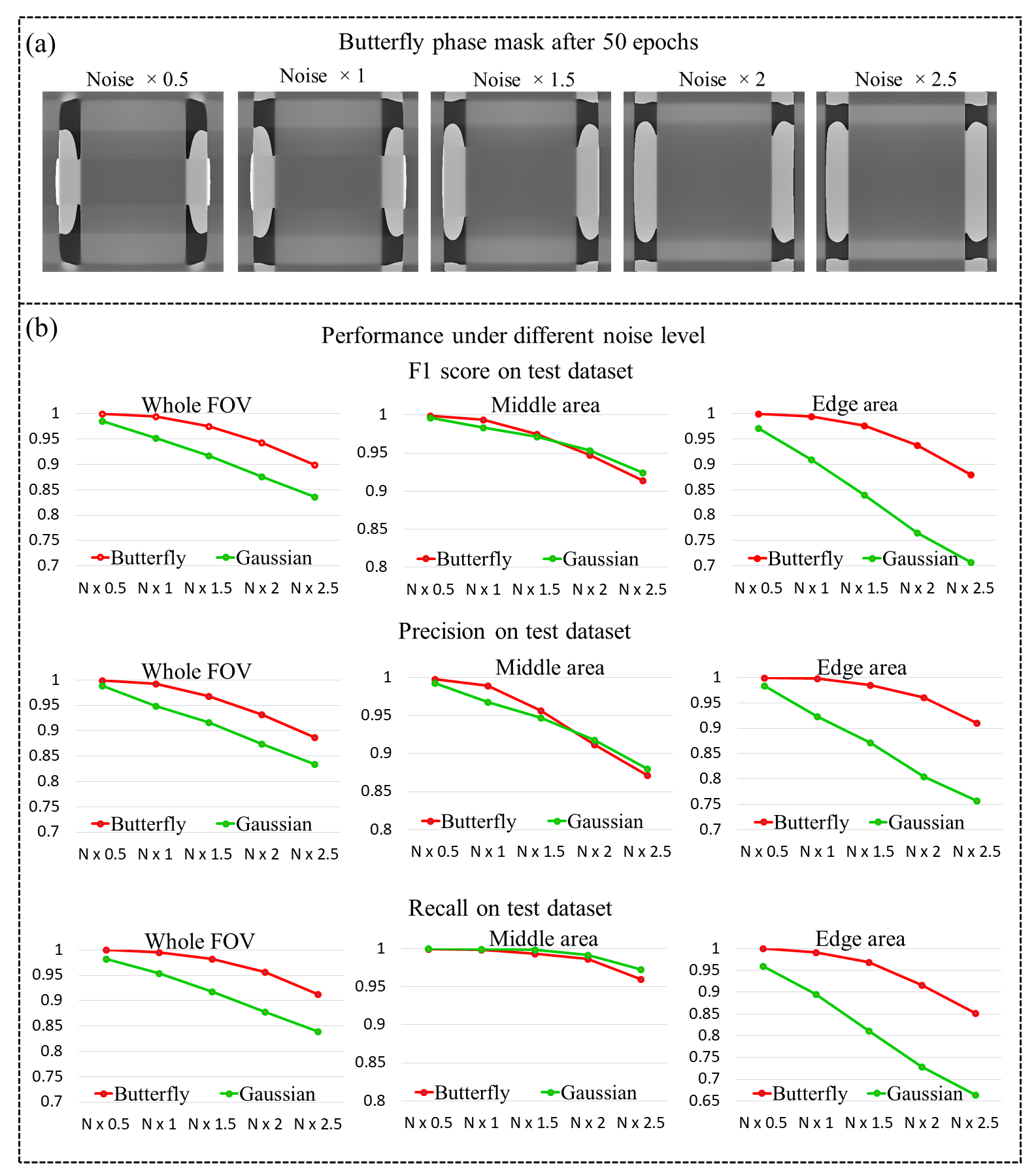

Supplement: Figs. S1 to S8 and Movies S1 and S2 [file NIHMS2009356-supplement-Figs__S1_to_S8_and_Movies_S1_and_S2.zip › S4.png]

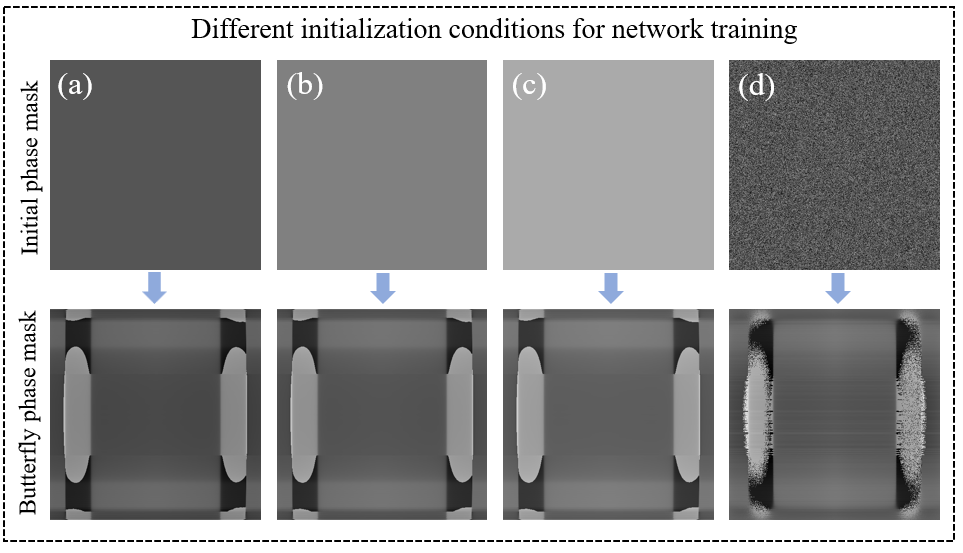

Supplement: Figs. S1 to S8 and Movies S1 and S2 [file NIHMS2009356-supplement-Figs__S1_to_S8_and_Movies_S1_and_S2.zip › S5.png]

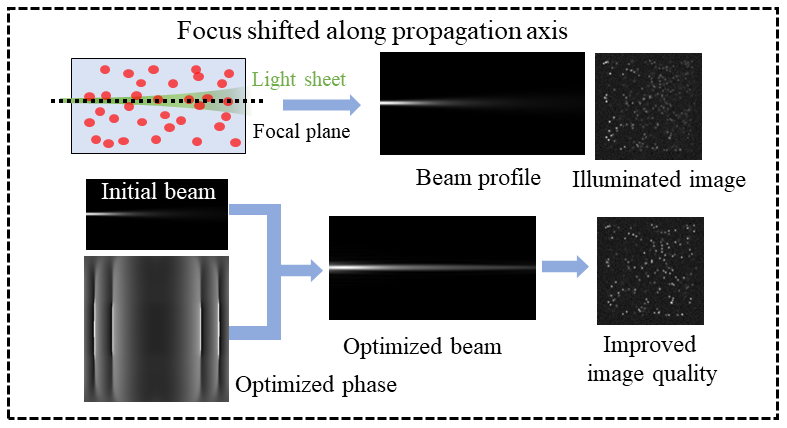

Supplement: Figs. S1 to S8 and Movies S1 and S2 [file NIHMS2009356-supplement-Figs__S1_to_S8_and_Movies_S1_and_S2.zip › S6.png]

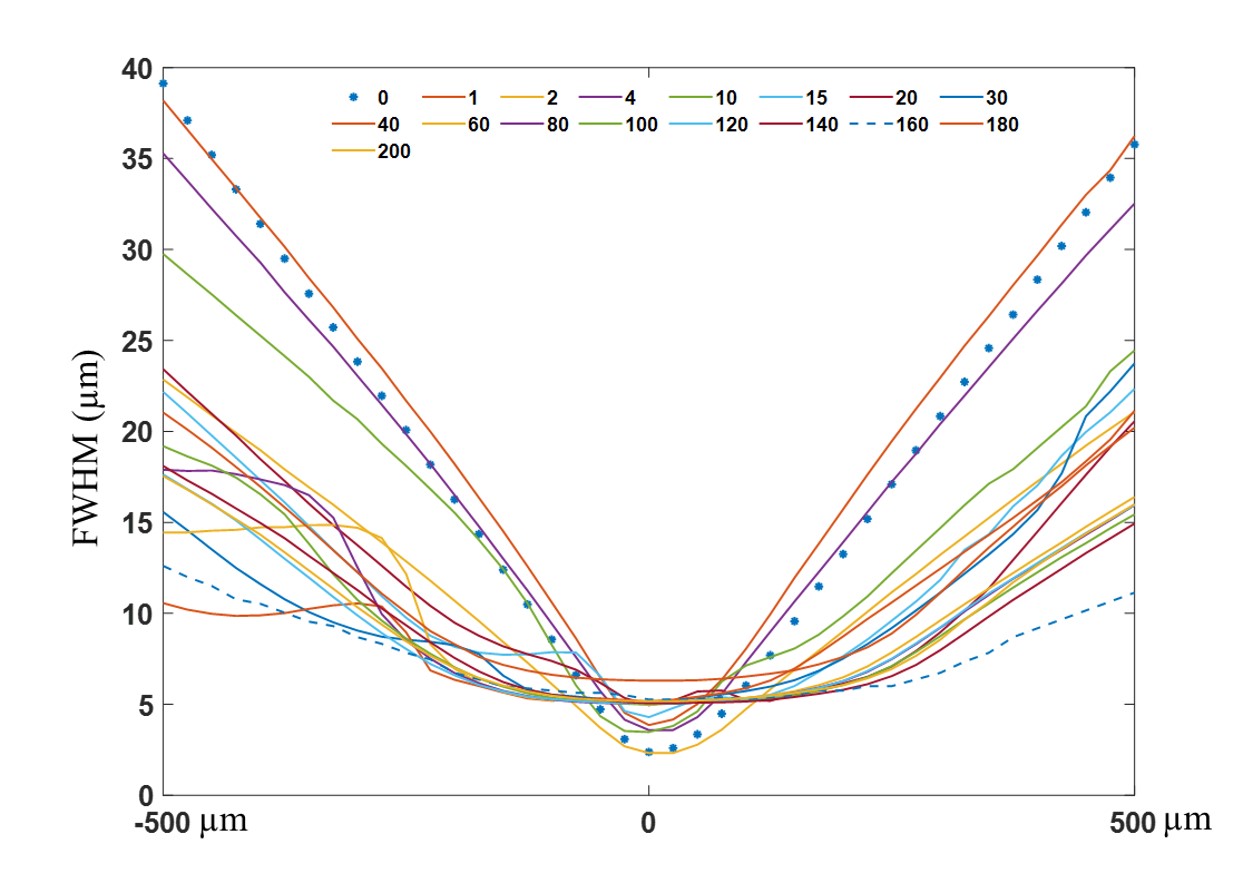

Supplement: Figs. S1 to S8 and Movies S1 and S2 [file NIHMS2009356-supplement-Figs__S1_to_S8_and_Movies_S1_and_S2.zip › S7.jpg]

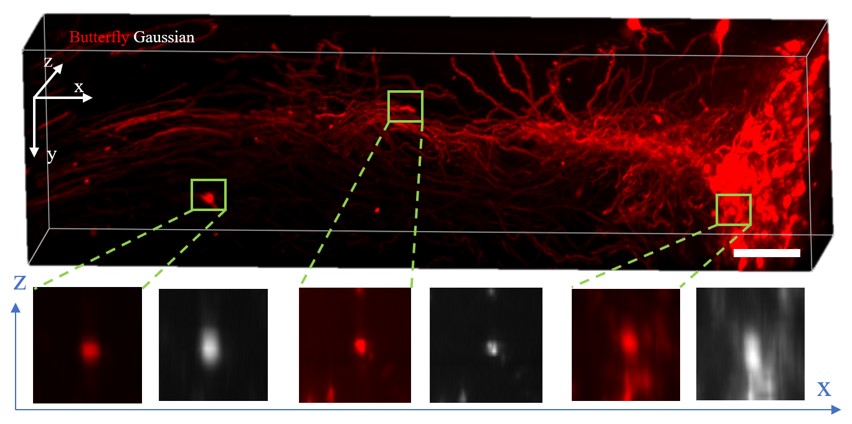

Supplement: Figs. S1 to S8 and Movies S1 and S2 [file NIHMS2009356-supplement-Figs__S1_to_S8_and_Movies_S1_and_S2.zip › S8.jpg]
